# [Hexane-2,5-dione bis­(thio­semi­carba­zon­ato)]nickel(II)

**DOI:** 10.1107/S1600536813012816

**Published:** 2013-05-15

**Authors:** Mohammad Safi Shalamzari, Atash V. Gurbanov, Seykens Heidic, Reza Kia, Shabnam Behrouzi

**Affiliations:** a Department of Pharmaceutical Sciences, University of Antwerp, Antwerp, Belgium; b Department of Organic Chemistry, Baku State University, Baku, Azerbaijan; c Department of Chemistry, University of Antwerp, Antwerp, Belgium; d Department of Chemistry, Science and Research Branch, Islamic Azad University, Tehran, Iran; e Deutsches Elektronen-Synchrotron (DESY), Division Structural Dynamics of (Bio)chemical Systems, Notkestrasse 85, 22607 Hamburg, Germany

## Abstract

In the title compound, [Ni(C_8_H_14_N_6_S_2_)], the Ni^II^ ion is coordinated by N_2_S_2_ donor atoms of the tetradentate thio­semicarbazone ligand, and has a slightly distorted square-planar geometry. In the crystal, inversion-related mol­ecules are linked *via* pairs of N—H⋯N and N—H⋯S hydrogen bonds, forming *R*_2_^2^(8) ring motifs. Mol­ecules are further linked by slightly weaker N—H⋯N, N—H⋯S and C—H⋯S hydrogen bonds, forming two-dimensional networks which lie parallel to the *bc* plane.

## Related literature

For standard values of bond lengths, see: Allen *et al.* (1987 ▶). For hydrogen-bond motifs, see: Bernstein *et al.* (1995 ▶). For related structures, see: Cowley *et al.* (2004 ▶); Lobana *et al.* (2011 ▶). The anti­tumor and anti­bacterial activity of thio­semicarbazones and thio­semicarbazides has been attributed to their ability to chelate trace metals, see: Kirschner *et al.* (1966 ▶). For the preparation of hexan-2,5-dionebis(thio­semicarbazone), see: Nandi *et al.* (1984 ▶).
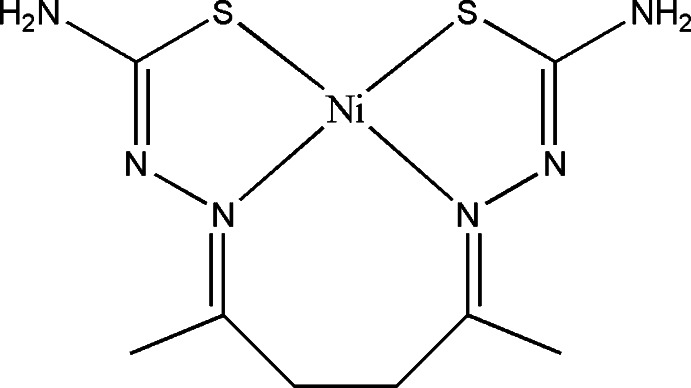


## Experimental

### 

#### Crystal data

[Ni(C_8_H_14_N_6_S_2_)]
*M* *_r_* = 317.08
Triclinic, 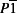

*a* = 7.8928 (3) Å
*b* = 8.0378 (3) Å
*c* = 11.0889 (4) Å
α = 69.720 (1)°
β = 75.214 (1)°
γ = 85.693 (1)°
*V* = 637.96 (4) Å^3^
*Z* = 2
Mo *K*α radiation
μ = 1.84 mm^−1^
*T* = 296 K
0.20 × 0.20 × 0.20 mm

#### Data collection

Bruker APEXII CCD diffractometer
Absorption correction: multi-scan (*SADABS*; Bruker, 2005 ▶) *T*_min_ = 0.711, *T*_max_ = 0.711
7275 measured reflections
3078 independent reflections
2833 reflections with *I* > 2σ(*I*)
*R*_int_ = 0.011

#### Refinement

*R*[*F* ^2^ > 2σ(*F* ^2^)] = 0.021
*wR*(*F* ^2^) = 0.059
*S* = 0.99
3078 reflections
156 parameters
H-atom parameters constrained
Δρ_max_ = 0.28 e Å^−3^
Δρ_min_ = −0.21 e Å^−3^

### 

Data collection: *APEX2* (Bruker, 2005 ▶); cell refinement: *SAINT-Plus* (Bruker, 2005 ▶); data reduction: *SAINT-Plus*; program(s) used to solve structure: *SHELXS97* (Sheldrick, 2008 ▶); program(s) used to refine structure: *SHELXL97* (Sheldrick, 2008 ▶); molecular graphics: *SHELXTL* (Sheldrick, 2008 ▶); software used to prepare material for publication: *SHELXTL* and *PLATON* (Spek, 2009 ▶).

## Supplementary Material

Crystal structure: contains datablock(s) global, I. DOI: 10.1107/S1600536813012816/su2598sup1.cif

Structure factors: contains datablock(s) I. DOI: 10.1107/S1600536813012816/su2598Isup2.hkl

Additional supplementary materials:  crystallographic information; 3D view; checkCIF report

## Figures and Tables

**Table 1:** Hydrogen-bond geometry (Å, °)

*D*—H⋯*A*	*D*—H	H⋯*A*	*D*⋯*A*	*D*—H⋯*A*
N3—H2*N*3⋯N2^i^	0.90	2.16	3.054 (2)	173
N3—H1*N*3⋯S1^ii^	0.90	2.58	3.4699 (17)	171
N6—H1*N*6⋯N2^iii^	0.90	2.28	3.1248 (19)	156
N6—H2*N*6⋯S2^iv^	0.92	2.67	3.5552 (16)	162
C3—H3*B*⋯S2^v^	0.96	2.87	3.7513 (17)	152

Symmetry codes: (i) 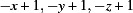; (ii) 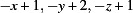; (iii) 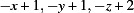; (iv) 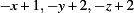; (v) 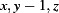.

**Fig. 1.**
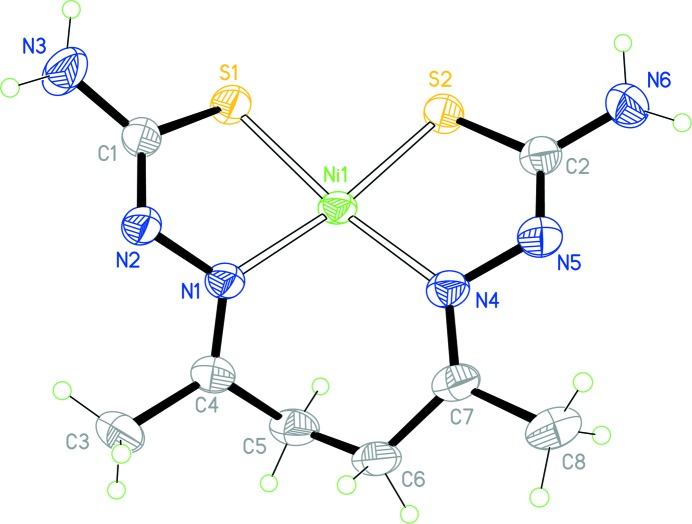
The molecular structure of the title compound, with atom labelling. Displacement ellipsoids are drawn at the 50% probability level.

**Fig. 2.**
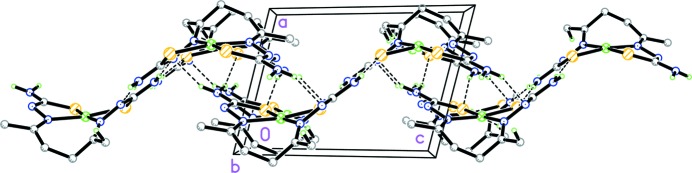
A view along the b-axis of the crystal packing of the title compound, showing the two-dimensional networks lying parallel to the bc plane. Only the H atoms involved in hydrogen bonding are shown.

## supplementary crystallographic information

### Comment

The antitumor and antibacterial of thiosemicarbazones and thiosemicarbazides have been attributed to their ability to chelate trace metals (Kirschner *et al.* 1966). Thiosemicarbazonato complexes are usually synthesized by the conventional approach of simply mixing alcoholic solutions of thiosemicarbazones and stoichiometric amounts of transition metal salt.

The asymmetric unit of the title compound, Fig. 1, comprises a thiosemicarbazone nickel(II) complex in which the Ni^II^ ion is coordinated by N_2_S_2_ donor atoms with a slightly distorted square-planar geometry. The angle between the mean planes S1–Ni1–N1 and S2–Ni1–N4 is 7.90 (4)°. The mean deviation of atom Ni1 from the mean plane N1–S1–S2–N4 is 0.0861 (5) Å. The bond lengths (Allen *et al.*, 1987) and angles are within the normal ranges and are comparable to those reported for related structures (Cowley *et al.* (2004); Lobana *et al.* (2011).

Pairs of intermolecular N—H···N and N—H···S hydrogen bonds make *R^2^_2_(8)* ring motifs (Bernstein *et al.*, 1995) [Table 1].

In the crystal, molecules are linked by N—H···N, N—H···S, and C—H···S interactions forming two-dimensional networks which lie parallel to the *bc* plane (Table 1 and Fig. 2).

### Experimental

Hexan-2,5-dionebis(thiosemicarbazone) was prepared by a method similar to that described by (Nandi *et al.* 1984). Hexan-2,5-dionebis(thiosemicarbazone) (1 mmol, 0.260 g) and nickel(II) acetate (0.66 g, 2.66 mmol) were placed in the main arm of a branched tube. Methanol was carefully added to fill the arms. The tube was sealed and immersed in an oil bath at 333 K while the branched arm was kept at ambient temperature. After 5 days, dark-red crystals (M.p. = 468 K) were isolated in the cooler arm and filtered off, washed with acetone and ether and dried in air (0.192 g; Yield 74%).

### Refinement

The N-bound H atoms were located in a difference Fourier map and constrained to refine on their parent atoms with U_iso_(H) = 1.2U_eq_(N). The C-bound H-atoms were included in calculated positions and treated as riding atoms: C—H = 0.93, 0.96 and 0.97 Å for CH, CH_3_ and CH_2_ H-atoms, respectively, with U_iso_(H) = k × U_eq_(C), where k = 1.5 for CH_3_ H-atoms, and = 1.2 for other H atoms.

### Crystal data

**Table:**

[Ni(C_8_H_14_N_6_S_2_)]	*Z* = 2
*M**_r_* = 317.08	*F*(000) = 328
Triclinic, *P*1	*D*_x_ = 1.651 Mg m^−^^3^
Hall symbol: -P 1	Melting point < 468 K
*a* = 7.8928 (3) Å	Mo *K*α radiation, λ = 0.71073 Å
*b* = 8.0378 (3) Å	Cell parameters from 4799 reflections
*c* = 11.0889 (4) Å	θ = 2.7–28.3°
α = 69.720 (1)°	µ = 1.84 mm^−^^1^
β = 75.214 (1)°	*T* = 296 K
γ = 85.693 (1)°	Prism, dark-red
*V* = 637.96 (4) Å^3^	0.20 × 0.20 × 0.20 mm

### Data collection

**Table:**

Bruker APEXII CCD diffractometer	3078 independent reflections
Radiation source: fine-focus sealed tube	2833 reflections with *I* > 2σ(*I*)
Graphite monochromator	*R*_int_ = 0.011
φ and ω scans	θ_max_ = 28.0°, θ_min_ = 2.0°
Absorption correction: multi-scan (*SADABS*; Bruker, 2005)	*h* = −10→10
*T*_min_ = 0.711, *T*_max_ = 0.711	*k* = −10→10
7275 measured reflections	*l* = −14→14

### Refinement

**Table:**

Refinement on *F*^2^	Primary atom site location: structure-invariant direct methods
Least-squares matrix: full	Secondary atom site location: difference Fourier map
*R*[*F*^2^ > 2σ(*F*^2^)] = 0.021	Hydrogen site location: inferred from neighbouring sites
*wR*(*F*^2^) = 0.059	H-atom parameters constrained
*S* = 0.99	*w* = 1/[σ^2^(*F*_o_^2^) + (0.0381*P*)^2^ + 0.1248*P*] where *P* = (*F*_o_^2^ + 2*F*_c_^2^)/3
3078 reflections	(Δ/σ)_max_ = 0.001
156 parameters	Δρ_max_ = 0.28 e Å^−^^3^
0 restraints	Δρ_min_ = −0.21 e Å^−^^3^

### Special details

**Table:**

Geometry. All esds (except the esd in the dihedral angle between two l.s. planes) are estimated using the full covariance matrix. The cell esds are taken into account individually in the estimation of esds in distances, angles and torsion angles; correlations between esds in cell parameters are only used when they are defined by crystal symmetry. An approximate (isotropic) treatment of cell esds is used for estimating esds involving l.s. planes.
Refinement. Refinement of F^2^ against ALL reflections. The weighted R-factor wR and goodness of fit S are based on F^2^, conventional R-factors R are based on F, with F set to zero for negative F^2^. The threshold expression of F^2^ > 2sigma(F^2^) is used only for calculating R-factors(gt) etc. and is not relevant to the choice of reflections for refinement. R-factors based on F^2^ are statistically about twice as large as those based on F, and R- factors based on ALL data will be even larger.

### Fractional atomic coordinates and isotropic or equivalent isotropic displacement parameters (Å^2^)

**Table:**

	*x*	*y*	*z*	*U*_iso_*/*U*_eq_	
Ni1	0.74078 (2)	0.65209 (2)	0.792634 (16)	0.02818 (7)	
S1	0.67960 (5)	0.84581 (4)	0.61696 (4)	0.03729 (9)	
S2	0.69288 (5)	0.85168 (4)	0.88575 (4)	0.03624 (9)	
N1	0.77048 (15)	0.49799 (14)	0.69038 (11)	0.0297 (2)	
N2	0.65361 (17)	0.51991 (15)	0.60798 (12)	0.0336 (2)	
N3	0.5029 (2)	0.73756 (19)	0.48368 (16)	0.0567 (4)	
H1N3	0.4658	0.8503	0.4602	0.068*	
H2N3	0.4647	0.6561	0.4575	0.068*	
N4	0.75863 (15)	0.48253 (15)	0.96705 (12)	0.0314 (2)	
N5	0.66994 (18)	0.53312 (16)	1.07736 (12)	0.0381 (3)	
N6	0.57514 (19)	0.76724 (18)	1.14636 (13)	0.0432 (3)	
H1N6	0.5353	0.6876	1.2274	0.052*	
H2N6	0.5298	0.8801	1.1246	0.052*	
C1	0.6069 (2)	0.68543 (18)	0.56807 (14)	0.0350 (3)	
C2	0.64152 (18)	0.70094 (18)	1.04654 (14)	0.0321 (3)	
C3	0.8992 (2)	0.2587 (2)	0.60870 (16)	0.0427 (3)	
H3A	0.8689	0.3227	0.5264	0.064*	
H3B	0.8197	0.1599	0.6581	0.064*	
H3C	1.0167	0.2163	0.5910	0.064*	
C4	0.88697 (18)	0.37854 (18)	0.68727 (14)	0.0321 (3)	
C5	1.01427 (19)	0.3582 (2)	0.77028 (16)	0.0389 (3)	
H5A	1.0445	0.4742	0.7680	0.047*	
H5B	1.1207	0.3055	0.7337	0.047*	
C6	0.9390 (2)	0.24249 (19)	0.91302 (16)	0.0376 (3)	
H6A	0.8753	0.1449	0.9115	0.045*	
H6B	1.0366	0.1917	0.9514	0.045*	
C7	0.82010 (18)	0.32434 (18)	1.00507 (15)	0.0336 (3)	
C8	0.7864 (2)	0.2088 (2)	1.14834 (17)	0.0457 (4)	
H8A	0.6655	0.2164	1.1913	0.069*	
H8B	0.8593	0.2480	1.1909	0.069*	
H8C	0.8131	0.0881	1.1543	0.069*	

### Atomic displacement parameters (Å^2^)

**Table:**

	*U*^11^	*U*^22^	*U*^33^	*U*^12^	*U*^13^	*U*^23^
Ni1	0.03387 (10)	0.02086 (9)	0.03506 (10)	0.00579 (6)	−0.01551 (7)	−0.01199 (7)
S1	0.0556 (2)	0.02174 (16)	0.04102 (19)	0.00748 (14)	−0.02323 (16)	−0.01184 (14)
S2	0.0531 (2)	0.02249 (16)	0.03655 (18)	0.00501 (14)	−0.01499 (15)	−0.01232 (13)
N1	0.0350 (6)	0.0238 (5)	0.0344 (6)	0.0044 (4)	−0.0139 (4)	−0.0118 (4)
N2	0.0452 (6)	0.0261 (5)	0.0369 (6)	0.0078 (5)	−0.0209 (5)	−0.0136 (5)
N3	0.0945 (12)	0.0322 (7)	0.0674 (9)	0.0223 (7)	−0.0587 (9)	−0.0231 (7)
N4	0.0343 (6)	0.0259 (5)	0.0382 (6)	0.0036 (4)	−0.0154 (5)	−0.0119 (5)
N5	0.0465 (7)	0.0305 (6)	0.0376 (6)	0.0068 (5)	−0.0131 (5)	−0.0111 (5)
N6	0.0557 (8)	0.0364 (7)	0.0375 (7)	0.0089 (6)	−0.0092 (6)	−0.0156 (5)
C1	0.0487 (8)	0.0267 (6)	0.0352 (7)	0.0080 (6)	−0.0181 (6)	−0.0132 (5)
C2	0.0335 (6)	0.0296 (6)	0.0371 (7)	0.0023 (5)	−0.0138 (5)	−0.0129 (5)
C3	0.0498 (9)	0.0348 (7)	0.0471 (8)	0.0102 (6)	−0.0098 (7)	−0.0219 (7)
C4	0.0337 (6)	0.0249 (6)	0.0372 (7)	0.0026 (5)	−0.0084 (5)	−0.0106 (5)
C5	0.0307 (6)	0.0355 (7)	0.0549 (9)	0.0079 (5)	−0.0151 (6)	−0.0190 (7)
C6	0.0402 (7)	0.0279 (7)	0.0519 (8)	0.0116 (6)	−0.0239 (6)	−0.0158 (6)
C7	0.0341 (6)	0.0259 (6)	0.0448 (7)	0.0034 (5)	−0.0200 (6)	−0.0105 (6)
C8	0.0503 (9)	0.0325 (7)	0.0487 (9)	0.0077 (6)	−0.0185 (7)	−0.0038 (6)

### Geometric parameters (Å, º)

**Table:**

Ni1—N1	1.9155 (11)	N6—H2N6	0.9237
Ni1—N4	1.9751 (12)	C3—C4	1.490 (2)
Ni1—S2	2.1542 (4)	C3—H3A	0.9600
Ni1—S1	2.1718 (4)	C3—H3B	0.9600
S1—C1	1.7434 (14)	C3—H3C	0.9600
S2—C2	1.7374 (15)	C4—C5	1.4922 (19)
N1—C4	1.2816 (18)	C5—C6	1.518 (2)
N1—N2	1.4181 (15)	C5—H5A	0.9700
N2—C1	1.3051 (17)	C5—H5B	0.9700
N3—C1	1.3392 (19)	C6—C7	1.496 (2)
N3—H1N3	0.9003	C6—H6A	0.9700
N3—H2N3	0.9003	C6—H6B	0.9700
N4—C7	1.2923 (17)	C7—C8	1.502 (2)
N4—N5	1.4202 (17)	C8—H8A	0.9600
N5—C2	1.2887 (18)	C8—H8B	0.9600
N6—C2	1.3619 (18)	C8—H8C	0.9600
N6—H1N6	0.8959		
			
N1—Ni1—N4	101.11 (5)	C4—C3—H3B	109.5
N1—Ni1—S2	171.94 (3)	H3A—C3—H3B	109.5
N4—Ni1—S2	86.61 (3)	C4—C3—H3C	109.5
N1—Ni1—S1	83.28 (3)	H3A—C3—H3C	109.5
N4—Ni1—S1	170.95 (4)	H3B—C3—H3C	109.5
S2—Ni1—S1	88.755 (14)	N1—C4—C5	117.00 (12)
C1—S1—Ni1	93.57 (5)	N1—C4—C3	123.93 (13)
C2—S2—Ni1	94.83 (5)	C5—C4—C3	119.06 (13)
C4—N1—N2	116.52 (11)	C4—C5—C6	111.47 (12)
C4—N1—Ni1	126.65 (10)	C4—C5—H5A	109.3
N2—N1—Ni1	116.82 (8)	C6—C5—H5A	109.3
C1—N2—N1	109.69 (11)	C4—C5—H5B	109.3
C1—N3—H1N3	119.4	C6—C5—H5B	109.3
C1—N3—H2N3	118.7	H5A—C5—H5B	108.0
H1N3—N3—H2N3	121.7	C7—C6—C5	118.83 (12)
C7—N4—N5	111.17 (12)	C7—C6—H6A	107.6
C7—N4—Ni1	133.49 (10)	C5—C6—H6A	107.6
N5—N4—Ni1	114.93 (8)	C7—C6—H6B	107.6
C2—N5—N4	113.32 (12)	C5—C6—H6B	107.6
C2—N6—H1N6	116.4	H6A—C6—H6B	107.0
C2—N6—H2N6	117.9	N4—C7—C8	122.59 (14)
H1N6—N6—H2N6	120.2	N4—C7—C6	123.82 (13)
N2—C1—N3	119.68 (13)	C8—C7—C6	113.50 (12)
N2—C1—S1	122.47 (11)	C7—C8—H8A	109.5
N3—C1—S1	117.82 (11)	C7—C8—H8B	109.5
N5—C2—N6	118.33 (13)	H8A—C8—H8B	109.5
N5—C2—S2	124.34 (11)	C7—C8—H8C	109.5
N6—C2—S2	117.25 (11)	H8A—C8—H8C	109.5
C4—C3—H3A	109.5	H8B—C8—H8C	109.5
			
N1—Ni1—S1—C1	26.73 (7)	Ni1—S1—C1—N3	159.93 (14)
S2—Ni1—S1—C1	−152.05 (6)	N4—N5—C2—N6	−172.75 (12)
N4—Ni1—S2—C2	−17.03 (6)	N4—N5—C2—S2	3.74 (18)
S1—Ni1—S2—C2	155.19 (5)	Ni1—S2—C2—N5	12.62 (13)
N4—Ni1—N1—C4	−46.03 (13)	Ni1—S2—C2—N6	−170.86 (11)
S1—Ni1—N1—C4	141.98 (12)	N2—N1—C4—C5	178.11 (12)
N4—Ni1—N1—N2	135.05 (9)	Ni1—N1—C4—C5	−0.80 (19)
S1—Ni1—N1—N2	−36.94 (9)	N2—N1—C4—C3	−3.2 (2)
C4—N1—N2—C1	−148.40 (13)	Ni1—N1—C4—C3	177.87 (11)
Ni1—N1—N2—C1	30.63 (15)	N1—C4—C5—C6	83.18 (16)
N1—Ni1—N4—C7	18.12 (14)	C3—C4—C5—C6	−95.57 (16)
S2—Ni1—N4—C7	−164.20 (13)	C4—C5—C6—C7	−82.08 (16)
N1—Ni1—N4—N5	−153.75 (9)	N5—N4—C7—C8	4.41 (19)
S2—Ni1—N4—N5	23.93 (9)	Ni1—N4—C7—C8	−167.68 (11)
C7—N4—N5—C2	165.09 (13)	N5—N4—C7—C6	−171.94 (13)
Ni1—N4—N5—C2	−21.23 (15)	Ni1—N4—C7—C6	16.0 (2)
N1—N2—C1—N3	176.82 (15)	C5—C6—C7—N4	9.3 (2)
N1—N2—C1—S1	−1.41 (18)	C5—C6—C7—C8	−167.36 (13)
Ni1—S1—C1—N2	−21.81 (14)		

### Hydrogen-bond geometry (Å, º)

**Table:**

*D*—H···*A*	*D*—H	H···*A*	*D*···*A*	*D*—H···*A*
N3—H2*N*3···N2^i^	0.90	2.16	3.054 (2)	173
N3—H1*N*3···S1^ii^	0.90	2.58	3.4699 (17)	171
N6—H1*N*6···N2^iii^	0.90	2.28	3.1248 (19)	156
N6—H2*N*6···S2^iv^	0.92	2.67	3.5552 (16)	162
C3—H3*B*···S2^v^	0.96	2.87	3.7513 (17)	152

Symmetry codes: (i) −*x*+1, −*y*+1, −*z*+1; (ii) −*x*+1, −*y*+2, −*z*+1; (iii) −*x*+1, −*y*+1, −*z*+2; (iv) −*x*+1, −*y*+2, −*z*+2; (v) *x*, *y*−1, *z*.
